# Modeling Fe(II) Complexes Using Neural Networks

**DOI:** 10.1021/acs.jctc.4c00063

**Published:** 2024-03-05

**Authors:** Hongni Jin, Kenneth M. Merz

**Affiliations:** † Department of Chemistry, Michigan State University, East Lansing, Michigan 48824, United States; ‡ Department of Biochemistry and Molecular Biology, Michigan State University, East Lansing, Michigan 48824, United States

## Abstract

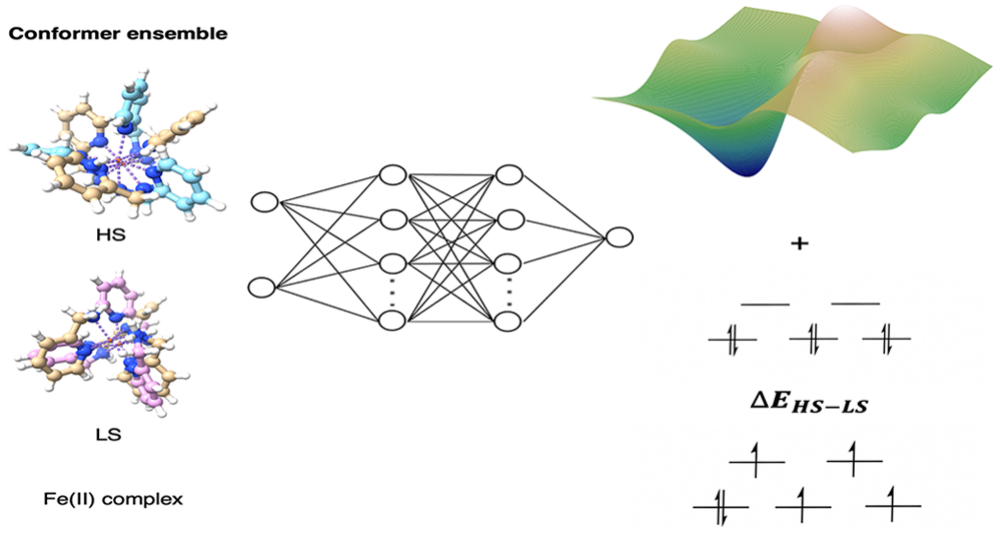

We report a Fe(II) data set of more than 23000 conformers in both low-spin (LS) and high-spin (HS) states. This data set was generated to develop a neural network model that is capable of predicting the energy and the energy splitting as a function of the conformation of a Fe(II) organometallic complex. In order to achieve this, we propose a type of scaled electronic embedding to cover the long-range interactions implicitly in our neural network describing the Fe(II) organometallic complexes. For the total energy prediction, the lowest MAE is 0.037 eV, while the lowest MAE of the splitting energy is 0.030 eV. Compared to baseline models, which only incorporate short-range interactions, our scaled electronic embeddings improve the accuracy by over 70% for the prediction of the total energy and the splitting energy. With regard to semiempirical methods, our proposed models reduce the MAE, with respect to these methods, by 2 orders of magnitude.

## Introduction

Transition metal complexes (TMCs) have many unique characteristics due to the fact that the transition metals from group 3 to group 12 have a range of oxidation states. The d valence shell actively interacts with charged and neutral ligands in transition metal chemistry because the d-orbitals are flexible enough to accommodate different types of ligands.^1^ One related property of TMCs is spin-crossover (SCO) where under the external stimulus of light, temperature perturbation, and pressure variation the spin state of TMCs can interconvert between the high-spin (HS) state and the low-spin (LS) state.^2−4^ The SCO complexes have promising applications in the field of sensors, memory storage, switches, the display industry, etc.^5−11^ TMCs with 3d^4^–3d^7^ electronic configurations are typical SCO complexes.^12^ But major efforts are still being made on Fe(II) complexes since they exhibit the most pronounced structural differences and they are also the most common examples in terms of SCO complexes.^13−15^ Iron(II), with 3d^6^ electronic configurations, has either the t_2g_^6^e_g_^0^ LS state or the t_2g_^4^e_g_^2^ HS state as the ground state. The spin splitting energy, *i.e*., the energy gap between both spin states, is usually within 10 kcal/mol.^12,16^ Such a relatively small energy difference requires accurate modeling methods to predict the true ground state. Highly accurate methods, like CASPT2 and MRCISD+Q^17,18^ can give reliable results; however, such computationally expensive methods can only be applied to small systems. An alternative, at the cost of losing some accuracy, is to use Density Functional Theory (DFT) methods.^19^ However, SCO complexes are sensitive to the exchange–correlation functional, which is the core part of DFT theory. Recent studies show that local functionals without HF exchange are typically biased toward the LS state, while hybrid functionals often favor the HS state.^20−23^

Another potential issue that has not been widely investigated is the effect of the geometry itself on the ground state of Fe(II) complexes. And much work on spin state energetics only considers a single geometry for each spin state.^12,14,16,24^ The results hold true only under these specific geometries since ligand conformations can cause different properties of TMCs.^25−29^ Most Fe(II) complexes exist as octahedral geometries in nature and have at least 2 unique ligands.^30^ These ligands interact with the central metal ion to stabilize the whole complex in a synergistic manner. The orientation of ligands in TMCs can even result in different types of interactions. For example, the small ligands CO and NO bind to Fe in the axial orientation, while the same ligands can also form weak noncovalent interactions in the parallel orientation.^31^ Such differences in orientation can result in large energy changes. Hence, both configurational and conformational effects of ligands should be considered to accurately predict the energetics of TMCs.

High-throughput screening is an efficient way to explore new functional molecules and materials.^32,33^ Usually thousands of candidates need to be identified and evaluated for target properties. And machine learning (ML) techniques have great potential to accelerate this process.^34^ With a well-trained model, the screening of thousands of candidates can be finished within seconds while keeping the accuracy of the reference method. To rapidly identify Fe(II) complexes with desirable properties, in this work we model the potential energy surface of Fe(II) complexes in both HS and LS states with ML. Neural network potentials (NNPs) have been widely investigated for organic molecules,^35−38^ while less work has been done for TMCs.^39−42^ However, to our best knowledge, none of these works have considered in detail the effect of ligand conformations on the energetics of TMCs. To achieve this, we first compile a set that includes both configurationally and conformationally diverse Fe(II) complexes and then use this data set to predict both the relative energy of conformers and the spin splitting energy accurately.

## Method

### Data Set

**Figure 1:**
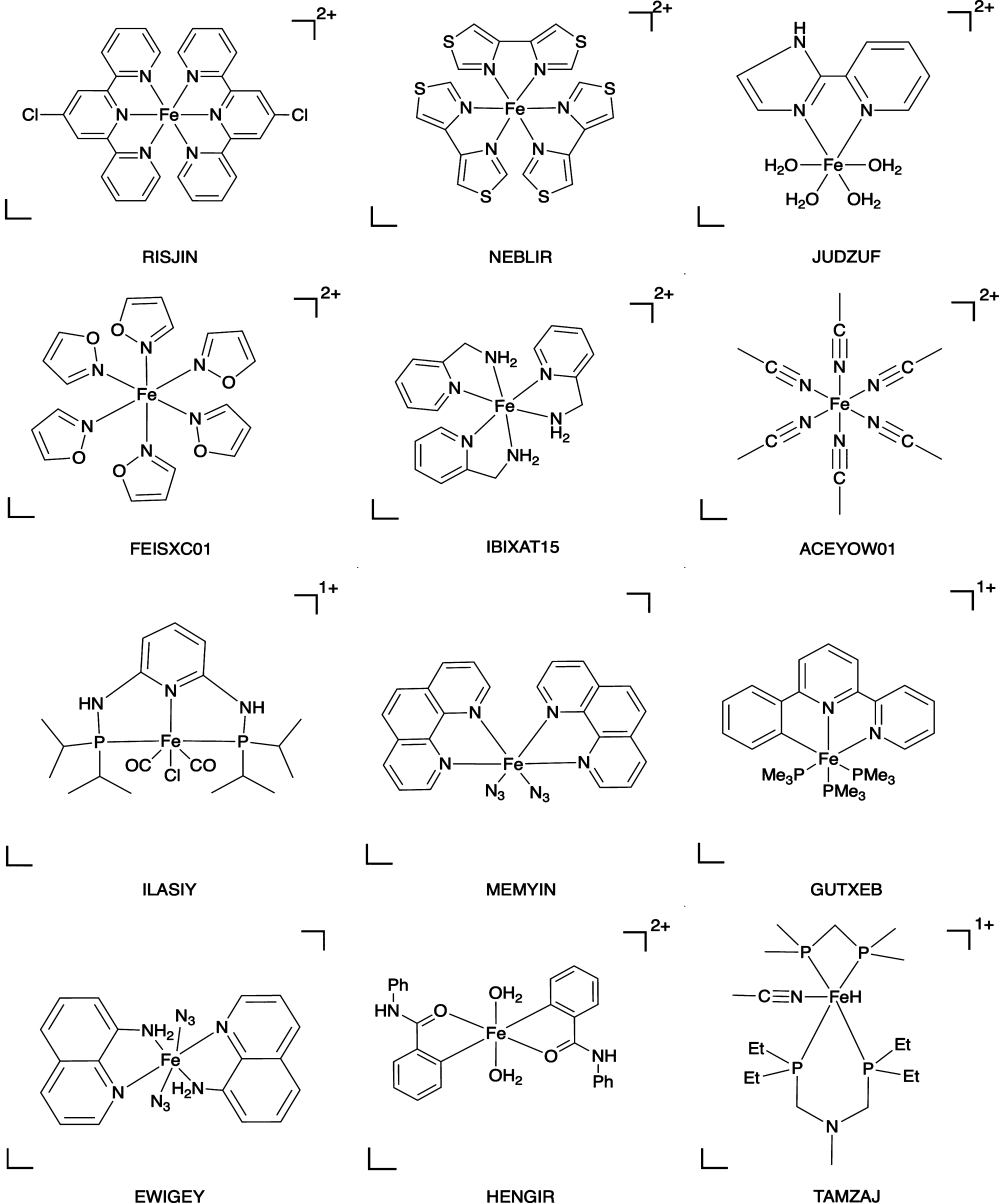
Typical structural examples with the refcode taken from the CSD in the Fe(II)_80 data set.

**Figure 2:**
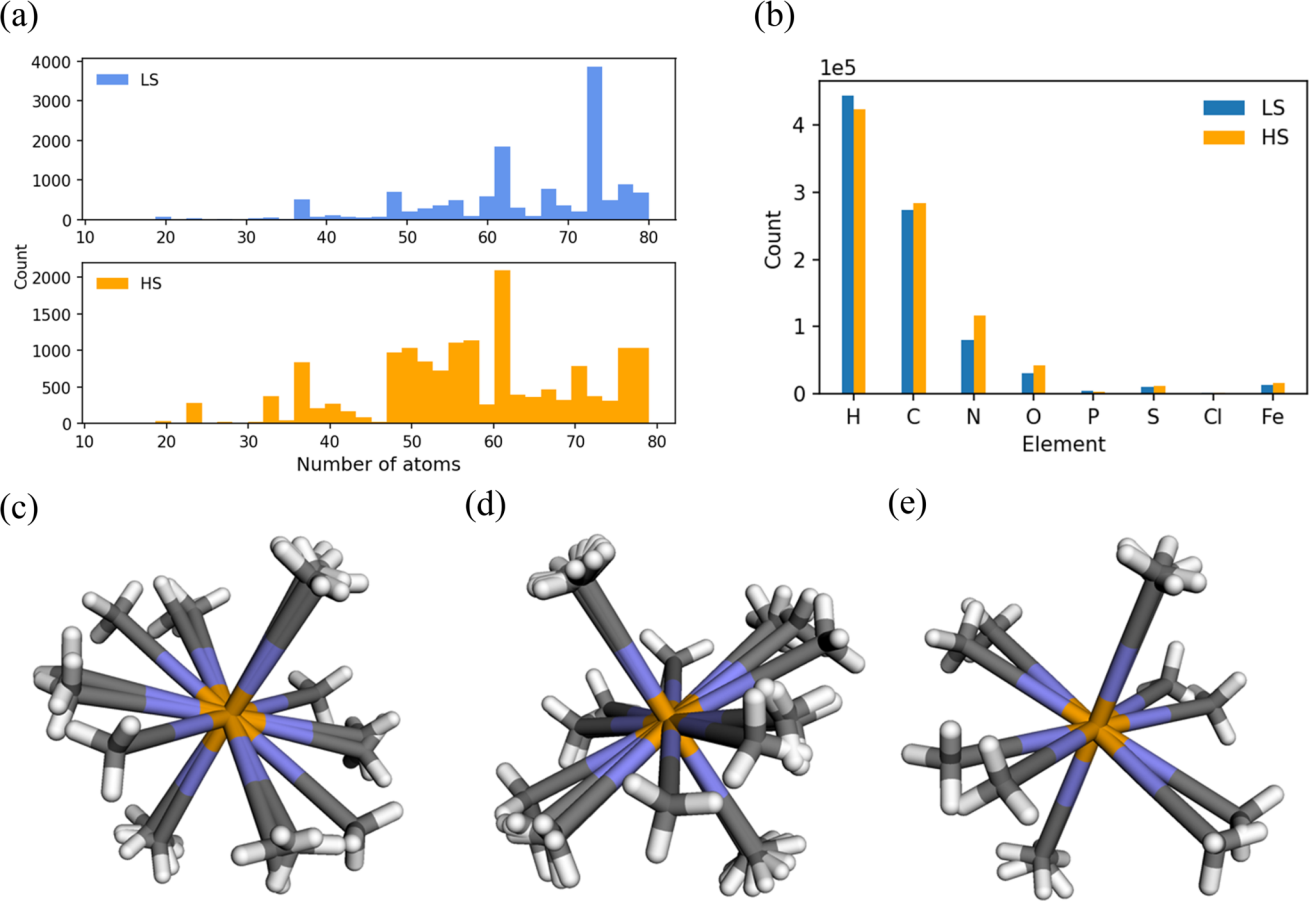
Chemical space in the Fe(II)_80 data set. (a) Molecular size distribution. (b) Element distribution. (c) Ensemble example of 3 conformers in HS spin state (refcode: ACEYOW01) (d) Ensemble example of 4 conformers in LS spin state (refcode: ACEYOW01). (e) Geometry with the lowest energy in HS and LS spin state. Δ*E*_HS–LS_ = 12.45 kcal/mol (refcode: ACEYOW01).

Nandy and co-workers^43^ reported a comprehensive data set which includes more than 240,000 crystallized mononuclear transition metal complexes (TMCs) from The Cambridge Structural Database (CSD).^44^ We followed their procedure^30^ to curate “computation-ready” complexes, i.e., both oxidation states and charges are already specified upon uploading without hydrogen atoms missing in the structures. Finally, we curated a subset of 383 unique Fe(II) well-defined complexes with 80 atoms or less. Various ligands and coordination patterns are covered in this set, as shown in Figure 1. Specifically, we assigned both HS state and LS state to each complex separately and then used the CREST^45^ package to generate spin-state-specific conformers. CREST uses metadynamics to cover a wider conformational space than traditional molecular dynamics simulations. Metadynamics has been shown previously to be a good sampling method in NNPs research.^46^ Conformers with minimal RMSD (≤0.1 Å) were removed. Each pair was aligned with respect to each other to get the optimum RMSD value.^47^ These crude geometries were further optimized using the B97-3c method.^48^ All optimizations were conducted using ORCA 5.0.4,^49^ with the *DEFGRID3*, *TightSCF*, *SlowConv*, and *SOSCF* settings. Geometries were excluded if (i) the optimization could not converge, (ii) an imaginary frequency was observed for the optimized geometry, or (iii) the deviation between the expected ⟨*Ŝ*^2^⟩ and the exact value was more than 1 μ_B_. This led to 15568 HS geometries and 13266 LS geometries to form the Fe(II)_80 data set (see Figure 2). Different DFT functionals may favor either the LS state or HS state depending on the design of the functional. The TPSSh functional^50^ was chosen as the reference method based on its robust performance over extensive tests.^12,13,51,52^ Final single-point energy calculations were conducted using the TPSSh-D4^53^ functional with the def2-TZVP^54^ basis set via ORCA 5.0.4 with the *TightSCF* setting. The RI-J approximation^55^ was used to accelerate the calculations with the def2/J^56^ auxiliary basis set. The Fe(II)_80 data set of 28834 geometries were randomly split into a training set (23834), a validation set (2500), and a test set (2500).

### Neural Networks

Most neural networks for 3D representations of molecules only consider the atom types and coordinates as the inputs. Such limited information, in our opinion, is not enough to differentiate spin states. In this work, we introduce charges and spin states into the SchNet^57^ model, a typical framework for message passing neural networks (MPNNs).^58^ The SchNet model includes message passing and update steps. In the message passing step, each neighbor of the central atom within a cutoff passes its information to the central atom via the *M*_*t*_ function which is designed by the neural network.
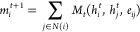
1where *h*_*i*_^*t*^ is the hidden representation of the central atom *i* at step *t*, *h*_*j*_^*t*^ is the hidden representation of the neighbor *j* at step *t*, and *e*_*ij*_ is the edge information between *i* and *j*, which is usually represented by the radial basis expansion of the relative position between atom *i* and atom *j.* Then *h*_*i*_^*t*^ is updated based on both *m*_*i*_^*t*+1^ and *h*_*i*_^*t*^
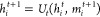
2where *U*_*t*_ is the Multilayer perceptron. The combination of message passing and an update at step *t* is called one interaction. Such interactions usually iterate several times so that the message can propagate among these atoms to better model the interactions of the whole system. In this work, the inputs of the model include the atom types, which are represented by the nuclear charge *Z*_*i*_ ∈ *N*, the Cartesian coordinates *r*_i_ ∈ *R*^3^, the total charge *Q* ∈ *Z*, and the spin state *S* ∈ *Z*. The nuclear charge *Z*, the total charge *Q*, as well as the spin state *S* are further transformed into high-dimensional features to get the final embeddings of each atom.

**Figure 3:**
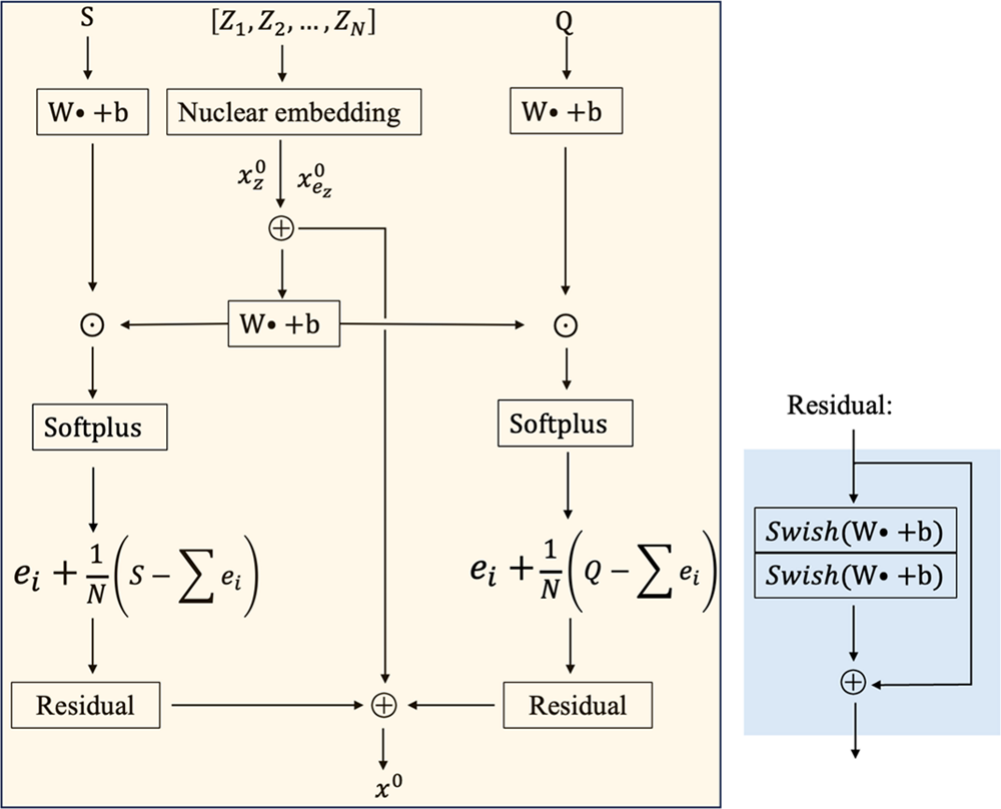
Schematic process for the complete embeddings *x*^0^ of a given molecule.

The representations of a given atom *x*^0^ ∈ *R*^*F*^ where *F* is the number of features include two parts: (i) the nuclear embeddings, *x*_N_^0^ = *x*_*z*_^0^ + *x*_*e*_*z*__^0^, where *x*_*z*_^0^ is the atom-type embeddings and *x*_*e*_*z*__^0^ is the atomic electron-configuration embeddings, both of which depend on the atom types; (ii) the electronic embeddings *x*_E_^0^ = *x*_Q_^0^ + *x*_S_^0^ where *x*_Q_^0^ is the charge embeddings and *x*_S_^0^ is the spin state embeddings. The atomic embeddings are defined as
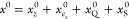
3where *x*_*z*_^0^ and *x*_*e*_*z*__^0^ are embedded via a look-up table based on the atom types. For *x*_Q_^0^ and *x*_S_^0^, SpookyNet^59^ uses the attention mechanism^60^ where three components including queries, keys, and values need to be well designed. In SpookyNet, one linear function is used to transform the nuclear embeddings into queries, then the charges are transformed into keys and values separately via two independent linear functions. The scaled dot product of queries and keys functions as the weight to differentiate the importance of each part in the charges. The values component is multiplied by the weight to obtain the final charge embeddings. The spin state embeddings follow the same process as the charge embeddings. Here, we simplify the mappings by only scaling the charge embeddings and spin state embeddings,
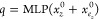
4
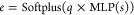
5
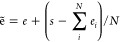
6
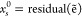
7where MLP is a Multilayer perceptron, Softplus is an activation function, *s* = Q for charge embeddings, and *s* = S for spin state embeddings. Finally, a residual block is used for a stable representation. The whole process for initializing the embeddings is given in Figure 3. Initially, the total charge of the complex is equally shared by each atom to obtain the partial charges. These partial charges are then multiplied by the nuclear embeddings to differentiate the importance of each partial charge. These partial charges are further scaled to make sure the sum of these partial charges equal to the total charge Q. The spin state follows the same process to obtain the spin state embeddings.

A main problem of current MPNN frameworks is that only interactions between pairs of atoms within the predefined cutoff are considered to simplify the computation. Long-range interactions are often ignored or calculated explicitly using standard physical forms.^35,59^ Recently, implicit long-range message passing models have shown promise in applications to organic molecules and periodic materials.^61,62^ For example, the Ewald-based message passing block transforms the features for long-range interactions in real space into frequency space using Fourier transforms. Such transformations take advantage of the fact that frequencies decay quickly thereby accelerating convergence. As a result, both short-range and long-range interactions can be summed up efficiently. The results show that Ewald message passing can reproduce the dispersion correction accurately. We refer readers to the original work^62^ for more details. Since the dispersion correction has non-negligible contributions to the total energy in TMCs, we also explored the use of Ewald message passing in this work.

We did several different types of comparisons. First, we tested whether the extra electron embeddings *x*_E_^0^ work in modeling TMCs. To achieve this, we compared three types of atomic embeddings, the attention-oriented electron embeddings proposed in SpookyNet, our scaled-embeddings and the pure nuclear embeddings, *i.e*., only *x*_*z*_^0^ which is the original inputs in the SchNet model. Second, the base model in this work is the SchNet model, but we also tested the PAINN^63^ model to figure out whether the extra vector representations are necessary if the target property is just invariant, i.e., the energy. Third, we compared these base models with the combined models, i.e., base model + Ewald message passing to understand whether the Ewald message passing can cover the long-range interactions in TMCs. Specifically, we tested it in two different ways: (i) the complete initial embeddings *x*^0^ as a whole are passed into the combined model, as proposed in the original work,^62^ the Ewald message passing is an independent block that can be added to any base model to form the combined model, and both models share the same embeddings throughout the iterations; (ii) the nuclear embeddings *x*_N_^0^ are passed to the base model, while the electron embeddings *x*_E_^0^ are passed to the Ewald message passing. In this case, both models are independently updated throughout the iterations.

### Training and Hyperparameters

All models use the 16 mini-batch size and the same initial learning rate of 5 × 10^–4^. All SchNet-based models use a milestone scheduler with 50000 warmup steps of a 0.2 warmup factor as well as a decay factor 0.1 at 150000, 25000, 350000 steps. And all PAINN-based models are trained using the AdamW optimizer with a weight decay λ = 0.01, and the plateau scheduler (decay factor 0.5 and patience 10) is also used to tune the training process.

## Results and Discussion

To evaluate the performance of these models, we first compared their ability to predict the total energy and the splitting energy. In our randomly split test set of 2500 Fe(II) conformers, 121 complexes have both HS and LS states, among which 1075 conformers are in the HS spin state while 654 conformers are in the LS spin state. We calculate the splitting energy (SE) for each pair from each complex, i.e., each pair includes a conformer of the HS state and a conformer of the LS state, but both conformers have the same configuration. Finally, 23446 pairs were retrieved from the test set. The mean absolute errors (MAE) in eV for both types of energies are given in Table 1.

**Table 1:** Mean Absolute Errors for the Total Energy and the Splitting Energy Predictions in eV^a^

	with electronic embeddings				without electron embeddings	
	SpookyNet_embeddings		Scaled_embeddings		only *x*_*z*_^0^	
model^b^	energy	Δ*E*_HS–LS_	energy	Δ*E*_HS–LS_	energy	Δ*E*_HS–LS_
SchNet	0.045	0.036	**0.037**	**0.030**	0.140	0.118
SchNet+EwaldMP	0.083	0.068	0.083	0.070	0.128	0.099
SchNet, EwaldMP	0.048	0.038	0.050	0.039		
PAINN	0.189	0.108	0.173	0.127	0.128	0.120
PAINN+EwaldMP	0.192	0.127	0.176	0.113	0.119	0.097
PAINN, EwaldMP	0.149	0.125	0.106	0.094		

a Best result in bold.

b The sign of “+” means the baseline model and the Ewald message passing share the same embeddings while “,” means the nuclear embeddings are fed into the baseline model and the electronic embeddings are inputs of the Ewald message passing.

The extra electronic embeddings *x*_E_^0^ greatly improve the performance of these models, and our scaled embeddings outperform the attention-oriented electronic embeddings in SpookyNet. For the SchNet baseline model, our scaled embeddings achieve the lowest MAE of 0.037 and 0.030 eV for the total energy and splitting energy, respectively, while the attention-oriented electronic embeddings yield a slightly worse MAE of 0.045 and 0.036 eV. Both types of embeddings make contributions to modeling the Fe complexes, since without them, the largest MAEs of 0.140 and 0.118 eV are obtained. Without the electronic embeddings *x*_E_^0^, the baseline PAINN model is slightly better than the SchNet model for the predictions of the total energy, with the MAE decreasing from 0.140 to 0.128 eV, while in terms of the splitting energy, both models yield a MAE of around 0.120 eV. Finally, if only *x*_*z*_^0^ is considered, in both types of baseline models, i.e., SchNet and PAINN, the baseline+EwaldMP decreases the MAE by around 0.01 and 0.02 eV for the total energy and splitting energy, respectively. For example, for the total energy, SchNet+EwaldMP achieves a MAE of 0.128 eV while the baseline SchNet yields a MAE of 0.140 eV. With the electronic embeddings *x*_E_^0^, simply adding the Ewald message passing to the baseline model as another contribution is not the best option for the Fe(II) data set. Since the electronic embeddings *x*_E_^0^ are already relevant to these long-range interactions, simply connecting two models together and sharing the same complete embeddings can cause the interactions to overlap. To circumvent this issue, these electronic embeddings *x*_E_^0^ should be fed into the Ewald message passing separately. As a result, the nuclear embeddings *x*_N_^0^ cover the short-range interactions, while the electronic embeddings *x*_E_^0^ reproduce the long-range interactions. For example, with the scaled embeddings, the MAE value of the total energy decreases from 0.083 eV (SchNet+Ewald) to 0.050 eV (SchNet, Ewald), along with the splitting energy error from 0.070 eV (SchNet+Ewald) to 0.039 eV (SchNet, Ewald). These comparisons indicate that the Ewald message passing approach can cover the long-range interactions well. But the most efficient way is to just feed the complete embeddings into the SchNet model. With the scaled embeddings, this baseline model can model the long-range interactions even better than the Ewald message passing at a reduced cost, giving the lowest MAE of 0.037 eV for the total energy as well as 0.030 eV for the splitting energy.

We also compared this ML based method with several semiempirical methods since the computational cost of all these methods is roughly at the same level. Recently, Hagen and co-workers designed the newly spin-polarized (sp)GFN*n*-xTB(*n* = 1,2)^64^ as an extension of the GFN*n*-xTB(*n* = 1,2) tight-binding methods to differentiate the spin states of TMCs. We also tested PM6-D3H4 as well as the PM7 method.^65−67^ (sp)GFN*n*-xTB(*n* = 1,2) calculations were conducted using *xtb*(68) version 6.6.1. The PM6-D3H4 and PM7 calculations were performed using MOPAC,^69^ version 22.0.6. All results are given in Table 2. We report the MAE of the splitting energy in eV as well as the number of correct spin states predicted as a qualitative analysis. In these semiempirical methods, some geometries were excluded due to job failures. In this extensive test, we found that the semiempirical methods did not predict the splitting energy nor the correct spin state very well. The splitting energy errors are consistent with the results tested on the TM90S benchmark set.^64^ In contrast, the SchNet model with the scaled embeddings only predicted 8 incorrect ground spin states with a MAE of 0.030 eV.

**Table 2:** Performance of the ML Model and All Tested Semiempirical Methods on the Spin State Splittings

	counts^b^	Δ*E*_HS–LS_^c^
SchNet^a^	23438/23446	0.030
PM6	6724/23307	2.8904
PM7	9757/23428	2.1062
spGFN1	5539/23428	3.5372
spGFN2	4407/23446	3.7195

a The SchNet baseline model with the scaled electronic embeddings is used as a comparison with these semiempirical methods.

b The number of correct spin states predicted. Since some systems could not run successfully in these semiempirical methods, the total numbers differ.

c The MAE value is given in eV.

## Conclusions

The minimal splitting energy of Fe(II) complexes makes them useful in applications in many fields. But it is challenging to carry out large-scale *in silico* screens for Fe(II) complexes due to the computational cost of the utilized QM methods. To predict the spin states and the total energy of Fe(II) complexes accurately and efficiently, we compiled a data set which covers both configurationally and conformationally diverse Fe(II) complexes in both HS and LS spin states. Next message passing neural networks were designed to model the potential energy surface of the resultant Fe(II) complexes. Our results indicate that our proposed scaled electronic embeddings cover long-range interactions implicitly and thus make good predictions for the total energy as well as the splitting energy. Moreover, they outperform alternative approaches like the Ewald message passing approach. Hence, our scaled electronic embedding approach is a valuable new addition to the model building tool kit. Finally, we show that this ML model greatly outperforms semiempirical methods in both qualitative and quantitative evaluations at lower cost. We anticipate that this model may be used to accelerate the *in silico* high-throughput screening of Fe(II) complexes for specific properties, like SCO.

## Data Availability

All data and code are available at https://github.com/Neon8988/Iron_NNPs.

## Supporting Information Available

Chemical space of the curated CSD complexes (PDF)
Curated CSD complexes used to generate conformations in this work (XLSX)
Semiempirical level energy of all geometries in the test set (XLSX)
