# Supplementary material for: Modeling Fe(II) Complexes Using Neural Networks
Source: J Chem Theory Comput. 2024 Mar 5;20(6):2551–8. doi: 10.1021/acs.jctc.4c00063 (PMC10976644; doi:10.1021/acs.jctc.4c00063)
Supplement: Supplementary file 1 — ct4c00063_si_001.pdf [file ct4c00063_si_001.pdf]

Supporting Information for Modeling Fe (II) Complexes Using Neural Networks

Hongni Jin[a] and Kenneth M. Merz, Jr.[a,b*]

[a]Department of Chemistry, Michigan State University,

East Lansing, Michigan 48824, United States

[b]Department of Biochemistry and Molecular Biology, Michigan State University,

East Lansing, Michigan 48824, United States

*Email: merz@chemistry.msu.edu

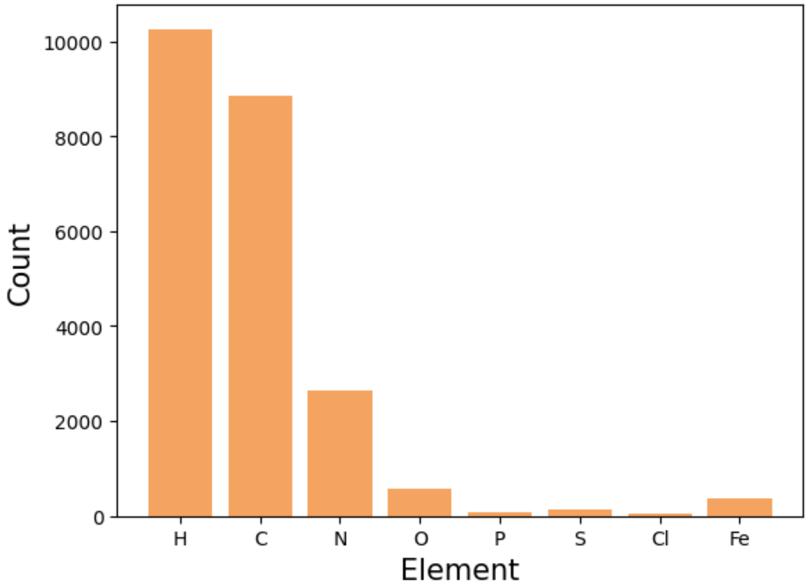

Figure S1. The element distribution in the 383 complexes.

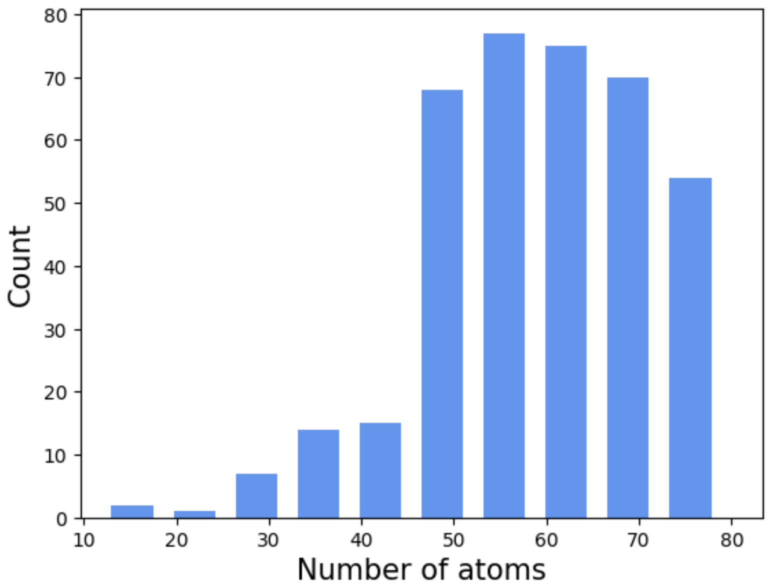

Figure S2. The molecular size of the 383 complexes.

Table S1. The denticity types of the 383 complexes.

Denticity type	Counts
6	51
5,1	53
4,2	15
4,1,1	27
3,3	115
3,2,1	1
3,1,1,1	15
2,2,2	27
2,2,1,1	35
2,1,1,1,1	4
1,1,1,1,1,1	40
